# Supplementary material for: UpStory: the uppsala storytelling dataset
Source: Front Robot AI. 2025 Jul 21;12:1547578. doi: 10.3389/frobt.2025.1547578 (PMC12320241; doi:10.3389/frobt.2025.1547578)

Post-Activity Questionnaire

ID: ____ Condition: ____ Date: _____________

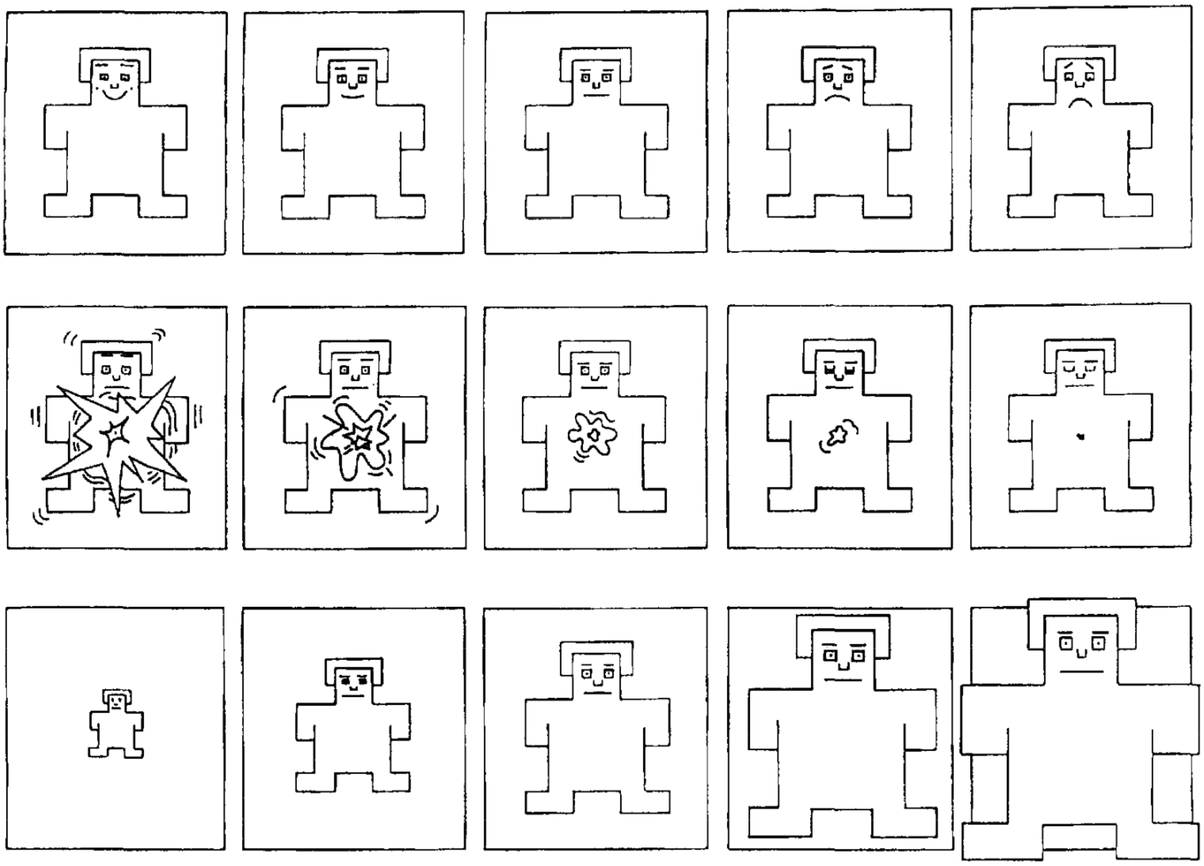

You and your best friend

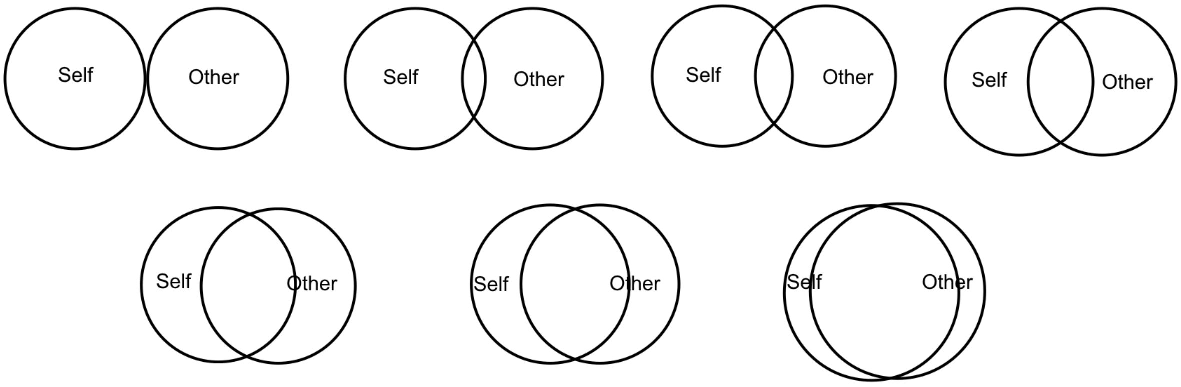

You and a bad guy

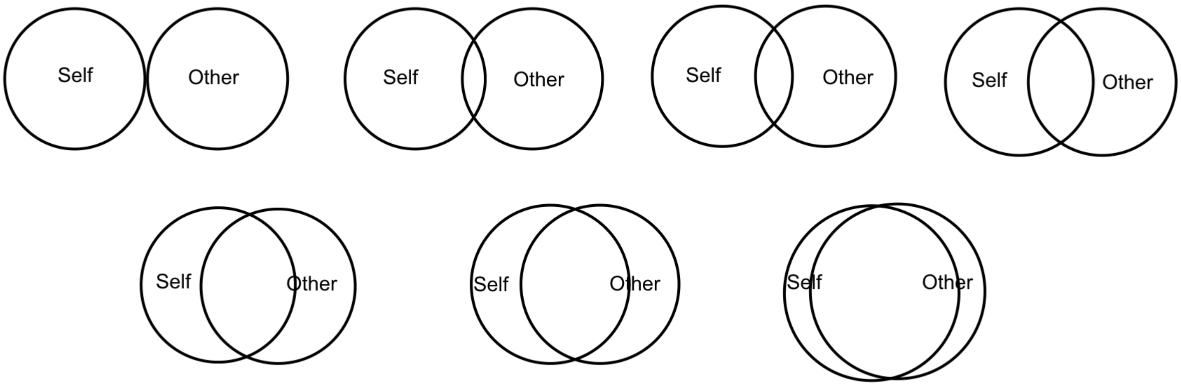

You and your partner

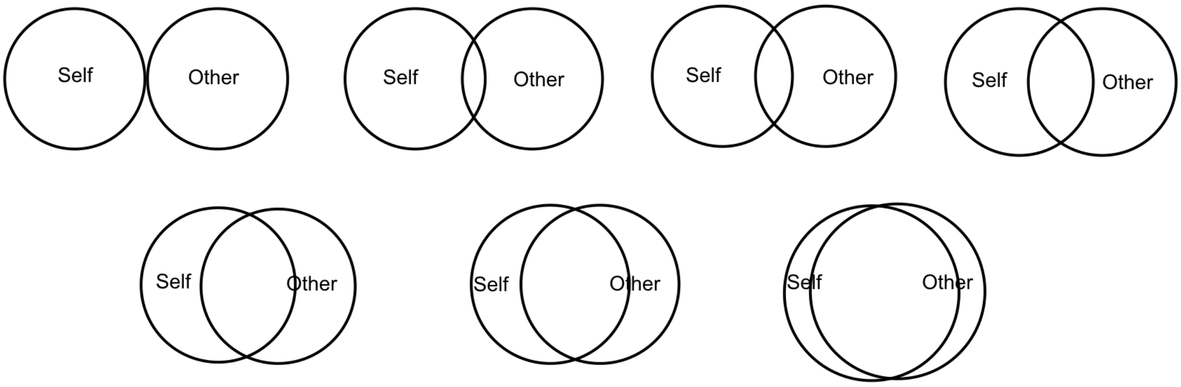

Supplement: Supplementary file 1 [file DataSheet2.pdf]
