# Supplementary figures and images for: UpStory: the uppsala storytelling dataset
Source: Front Robot AI. 2025 Jul 21;12:1547578. doi: 10.3389/frobt.2025.1547578 (PMC12320241; doi:10.3389/frobt.2025.1547578)

# Pre-Activity Questionnaire

ID: \_\_\_\_\_

Condition: \_\_\_\_\_

Date: \_\_\_\_\_

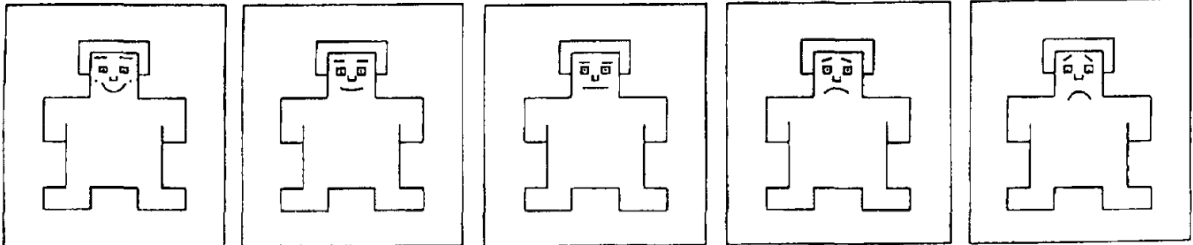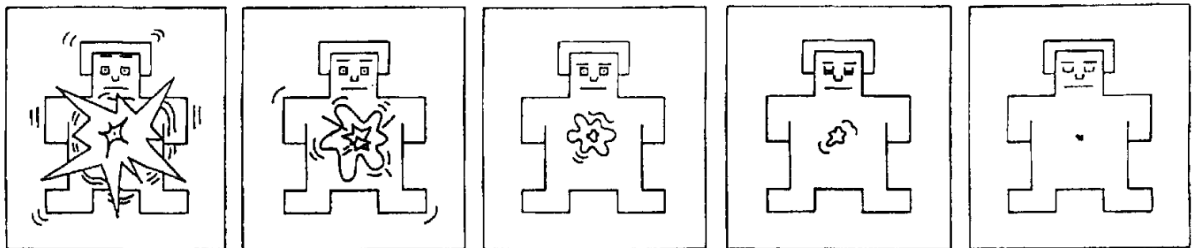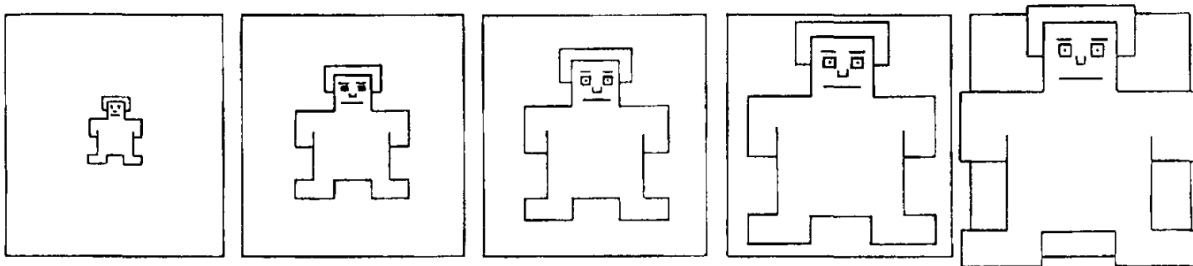

Supplement: Supplementary file 2 [file DataSheet3.pdf]
