# Supplementary material for: UpStory: the uppsala storytelling dataset
Source: Front Robot AI. 2025 Jul 21;12:1547578. doi: 10.3389/frobt.2025.1547578 (PMC12320241; doi:10.3389/frobt.2025.1547578)
Supplement: Supplementary file 3 [file DataSheet1.pdf]

ID: _______

# My closest friends are
